# Survey Modalities and COVID-19 Vaccine Uptake in Vietnamese Americans: Cross-Sectional Study

**DOI:** 10.2196/77520

**Published:** 2026-02-25

**Authors:** Celine Nguyen, Alexander Le, Paul Yeh, Ben King, Angelica Nguyen, Jannette Diep, Bich-May Nguyen

**Affiliations:** 1 The University of Texas Southwestern Medical Center, Dallas, TX, United States; 2 College of Medicine, Texas A&M University, College Station, TX, United States; 3 Tilman J Fertitta Family College of Medicine, University of Houston, 5055 Medical Circle, Houston, TX, 77204-6064, United States, 1 713 743-8514; 4 Boat People SOS, Houston, TX, United States

**Keywords:** health disparities, immigrant health, population health, public health, paper survey, methodology

## Abstract

**Background:**

Diverse survey methodologies are essential to ensure equitable representation in public health research, particularly among minority populations. This study evaluates demographic differences among Vietnamese Americans who completed paper versus electronic surveys while administering the National Institutes of Health Community Engagement Alliance Common Survey 2, which focused on COVID-19–related topics.

**Objective:**

The study aimed to (1) describe the sociodemographic characteristics of survey respondents; (2) compare paper versus electronic survey modalities and their associations with respondent demographics; and (3) highlight the role of community-based organizations (CBOs) and community-engaged research in improving representativeness and inclusivity.

**Methods:**

Vietnamese adults in Texas were recruited in two phases. In phase 1 (September 2021-March 2022), surveys were administered online. In response to CBOs’ feedback, phase 2 (December 2022-April 2023) added paper surveys administered by bilingual recruiters; surveys were available in English and Vietnamese. Descriptive analyses were conducted for both phases. Multivariate logistic regression, limited to phase 2, assessed factors associated with survey method preferences, including language, sex, education, COVID-19 history, and willingness to participate in COVID-19 trials.

**Results:**

Phase 1 included 224 electronic surveys. Phase 2 included 359 surveys (electronic: n=124, 34.5% and paper: n=235, 65.5%). Vietnamese speakers were significantly more likely to complete paper surveys (adjusted odds ratio [AOR] 100.9, 95% CI 24.3‐418.9; *P*<.001), as were female participants (AOR 5.09, 95% CI 1.43‐18.1; *P*=.01). Conversely, those with a history of COVID-19 (AOR 0.16, 95% CI 0.05‐0.52; *P*=.002), a college or higher education level (AOR 0.18, 95% CI 0.05‐0.67; *P*=.01), and high willingness to participate in COVID-19 trials (AOR 0.21, 95% CI 0.06‐0.81; *P*=.02) were less likely to complete paper rather than electronic surveys.

**Conclusions:**

Incorporating paper surveys and engaging CBOs improved participation among Vietnamese speakers and those without postsecondary education, addressing the underrepresentation observed in phase 1. These findings highlight the importance of tailored survey methodologies to achieve demographic inclusivity in public health research.

## Introduction

Historically marginalized populations are often underrepresented in public health research, which can exacerbate existing health disparities by failing to capture the unique health challenges these groups face adequately. Among these populations, the Vietnamese American community presents distinctive challenges and opportunities for researchers. Despite significant progress in medical advancements, Asian Americans, including Vietnamese Americans, continue to experience disparities in health outcomes, largely driven by nonmedical drivers of health such as language accessibility, cultural differences, and varying levels of trust in public institutions [1]. These disparities are further compounded by the limited representation of Asian Americans in public health research, which often relies on online surveys that may not fully engage this population [2-5].

Survey methodology is crucial in public health research, especially when engaging economically disadvantaged populations. While online surveys are increasingly used for convenience and broad reach, they may not be as effective for populations with limited digital access or health literacy, such as older Vietnamese Americans [6-8]. For example, one study conducted in Chicago found that disparities in digital access were associated with race, ethnicity, and income [9]. In contrast, paper surveys provide an accessible alternative that may enhance participation among these groups and potentially lead to more representative data collection [10]. However, the comparative effectiveness of paper surveys versus electronic ones in accurately reaching and representing Vietnamese Americans remains underexplored, indicating a need for further research in this area [11,12].

Vietnamese Americans face several specific health disparities, including higher rates of chronic conditions such as hepatitis B, cancer [13-17], and non–small cell lung cancer [18] compared to other racial and ethnic groups. The historical context of migration, coupled with socioeconomic challenges, has also led to significant mental health issues within this community, with elevated rates of posttraumatic stress disorder and depression among refugees and their descendants [19-21]. However, these health issues are frequently underreported and understudied, partly due to the barriers hindering their research participation [22,23]. Vietnamese Americans, particularly older adults and recent immigrants, have a high level of non-English language preference, which restricts their ability to engage with research materials that are not available in their native language [24-26]. Additionally, there is often a deep-seated mistrust in public institutions among Vietnamese Americans, stemming from historical experiences of political oppression and displacement, which can further deter participation in research conducted by these institutions [27-30].

Community-based organizations (CBOs) have long been recognized as vital partners in addressing these challenges and promoting equity in public health research [31,32]. Research has shown that CBOs can increase participation rates and data accuracy by leveraging their established relationships within these communities [33]. Community-engaged research (CEnR) emphasizes actively involving community organizations, stakeholders, and members in the research process to ensure that studies are culturally relevant and inclusive [34-36]. CBO involvement in CEnR is particularly important in studies involving populations with specific cultural and linguistic needs, as they can enhance the reliability and representativeness of the collected data [33].

In response to the pressing need to address research gaps among communities of color during the COVID-19 pandemic, the National Institutes of Health (NIH) launched the Community Engagement Alliance (CEAL) Against COVID-19 in Disproportionately Affected Communities Consortium. This initiative aimed to conduct CEnR and outreach in collaboration with local entities. As part of the Texas CEAL initiative, our team fielded the Common Survey 2 instrument twice to examine the impact of the pandemic on Vietnamese American adults in Texas. During the first year, data were collected exclusively through electronic surveys [37,38], while in the second year, a mixed approach incorporating both electronic and paper surveys was used in response to CBO feedback. This study compares participant demographics across these two survey modalities to evaluate how different data collection strategies influence engagement and representation. Additionally, it examines COVID-19–related challenges and explores facilitators and barriers to vaccine uptake, providing valuable insights into the role of social determinants of health in shaping health behaviors and outcomes in this population.

This study had three objectives: (1) to describe the sociodemographic characteristics of Vietnamese American respondents; (2) to compare paper versus electronic survey modalities and evaluate their associations with respondent demographics; and (3) to highlight the role of CBOs and CEnR in enhancing representativeness and inclusivity in minority population health research. These findings aim to inform the development of equitable interventions and policies that address nonmedical drivers of health, ultimately contributing to the reduction of health disparities among Vietnamese Americans and other marginalized communities.

## Methods

### Study Design and Population

As part of the NIH CEAL initiative, we used the Common Survey 2 instrument in this repeated cross-sectional survey approved by the Institutional Review Board at the University of Houston (June 4, 2021; STUDY00003046).

The CEAL Common Survey 2 is a 23-item questionnaire designed to comprehensively assess COVID-19–related attitudes, behaviors, and experiences. This instrument containing questions pertaining to vaccine hesitancy, adherence to preventive measures, and willingness to participate in clinical trials has been described previously [27]. The CEAL Common Survey 2 has been widely implemented across CEAL-affiliated projects but has not undergone formal psychometric validation, such as reliability or construct validity testing [39]. This reflects the program’s rapid launch during the COVID-19 public health emergency in September 2020, when urgent deployment precluded full validation procedures. Despite this limitation, the survey has been applied across diverse communities and study contexts, supporting its use as a standardized, though not formally validated, instrument. The English and Vietnamese versions are provided in MultimediaAppendices 1  2.

A translator from a collaborating CBO translated the survey from English into Vietnamese. Subsequently, a different translator from the same CBO performed a back-translation of the survey from Vietnamese to English. To ensure clarity and cultural relevance for the target population, native Vietnamese speakers at another CBO reviewed and reconciled the translations. The survey and consent form were accessible online in English and Vietnamese. On average, participants required 15 minutes to complete the survey in English and 20 minutes in Vietnamese [27].

The study design was divided into two phases. Phase 1, conducted between September 2021 and March 2022, exclusively used an online survey format. However, recognizing the limitations of an online-only approach in response to the CBO feedback and preliminary analyses from phase 1 and phase 2 (December 2022-April 2023) incorporated a paper-based survey option in addition to online recruitment.

Participants were eligible for inclusion in the study if they met the following criteria: they were at least 18 years of age, self-identified as being of Vietnamese heritage, resided in the state of Texas, and were proficient in reading and writing either English or Vietnamese.

### Recruitment

A convenience sampling method was used to recruit Vietnamese Americans for the study. Data collection in phase 1 was primarily facilitated through digital channels, capitalizing on the existing networks of local and national Vietnamese American civic groups, religious organizations, and networks on social media platforms.

The survey remained open for responses from September 20, 2021, to March 4, 2022. Recruitment efforts were conducted virtually, in both English and Vietnamese, through email distribution lists through the Vietnamese American Medical Association, Vietnamese American Nurses Association, Vietnamese American Pharmacists Association, the Progressive Vietnamese American Organization, and the Vietnamese Culture and Science Association; social media platforms such as Facebook groups; an online webinar hosted by Boat People SOS—Houston (BPSOS); and an internet advertisement placed on the Google Marketing Platform that ran from January 6 to 23, 2022. Additional recruitment was conducted in person with recruitment flyers in both English and Vietnamese with QR codes at BPSOS literacy classes and two health fairs organized by Vietnamese American Nurses Association, Vietnamese American Medical Association, and Vietnamese American Pharmacists Association. Furthermore, the recruitment flyers were displayed at two health clinics in southwest Houston serving predominantly Vietnamese populations. Participants who completed the survey and provided a valid email address or phone number were entered into a raffle to win one of five US $50 gift cards [27].

Phase 2 used the same electronic recruitment methods from phase 1 and expanded survey administration to include simultaneous paper survey distribution. These surveys remained open for responses from December 26, 2022 to April 30, 2023. Paper surveys were strategically distributed at community events and health fairs led by researchers and local CBO-trained bilingual community members who recruited participants at a local clinic in southwest Houston with a large Vietnamese American patient population and CBO events sponsored by the organizations above. Trained community members used the paper surveys and did not offer electronic participation. Research team members performed double data entry of paper survey responses into a separate online survey. All entries were compared for accuracy and quality assurance. Unclear or inconsistent responses underwent a structured review process, with discrepancies addressed through a consensus-driven decision-making process.

### Measures

#### Dependent Variable

The primary outcome of interest in this study was the mode of survey completion, categorized as either electronic or paper-based. This reflects both a participant’s opportunity to encounter the survey in a given format and their decision to complete it. Differences in where and how each modality was used, such as online recruitment versus CBO events, are therefore embedded in this outcome and influence interpretation of participation patterns. Participants who completed the survey online were classified under the “electronic survey” group, while those who completed a paper version were assigned to the “paper survey” group.

#### Independent Variables

Sociodemographic characteristics were assessed to examine differences among respondents based on the mode of survey completion. Gender was categorized as female, male, other, or prefer not to answer. Age was recorded as a continuous variable and further categorized into the following age groups: 18‐24, 25‐34, 35‐44, 45‐54, 55‐64, 65‐74, and ≥75 years. Sexual orientation was classified as heterosexual, homosexual, other, or prefer not to answer. Language preference was determined based on participants’ self-reported preferred language for survey completion (English or Vietnamese). Educational attainment was recorded as a college degree or higher versus less than a college degree. Insurance status was classified as insured or uninsured.

In addition to sociodemographic characteristics, health and COVID-19–related variables were included in the analysis. Participants reported their history of prior COVID-19 infection (yes, no, or unsure diagnosis). COVID-19 vaccination status was categorized as fully vaccinated, first dose of a 2-dose vaccine, not vaccinated, or prefer not to answer.

To assess trust in health care and research institutions, participants rated their level of trust in various sources, including the NIH, health care providers, university hospitals, community clinics, pharmaceutical companies, and family or friends. Response options included a great deal, a fair amount, not very much, not at all, or no opinion. Trust was dichotomized into trust (a great deal or a fair amount) versus no trust (not very much or not at all). Willingness to participate in COVID-19 clinical trials was measured using a 7-point Likert scale. For analysis, responses were categorized into high willingness (scores of 6 or 7 on the scale) versus less than high willingness (≤5 on the scale).

To evaluate health literacy and access to health care information, participants responded to the question, “How often do you need someone to help you read written information from your doctor or drug store?” Response options included always, often, sometimes, rarely, never, or prefer not to respond. For analysis, responses were categorized as requiring assistance if participants selected always, often, or sometimes.

The independent variables included in regression models, including language, education, trust, COVID-19 history, and willingness to participate in trials, were selected based on prior CEAL studies [27] and conceptual frameworks identifying social determinants of health and community trust as key drivers of health engagement during the COVID-19 pandemic. These constructs were prioritized given their centrality to CEAL program aims and their relevance in understanding disparities in research participation among Vietnamese Americans.

### Statistical Analysis

We analyzed data using STATA (version 17; StataCorp LLC). Multivariate logistic regression models were used to explore the factors associated with the choice of survey mode in phase 2. Phase 1 data were not included in these models because all respondents in that phase completed the survey electronically, resulting in no variability with respect to survey modality. Although combining electronic data from both phases could have increased statistical power, this approach was not pursued due to meaningful contextual differences between phases, including the period of data collection, the recruitment strategies used, and the introduction of CBO-led in-person events during phase 2. Restricting analyses to phase 2, therefore, ensured analytic consistency and reduced the potential for bias introduced by structural differences between phases. These models adjusted for key demographic and socioeconomic variables, including age, gender, language preference (English or Vietnamese), and education level. In addition, the models adjusted for key constructs about COVID-19 included in the survey, such as history of COVID-19 infection, COVID-19 vaccination history, trusted sources of health information, and willingness to participate in COVID-19 clinical trials. The logistic regression models generated adjusted odds ratios (AOR) and 95% CIs for each predictor variable, with statistical significance determined by a 2-sided *P* value threshold of *P*<.05.

### Ethical Considerations

The study was conducted according to the guidelines of the Declaration of Helsinki and approved by the institutional review board of the University of Houston (STUDY00003046; June 4, 2021).

Informed consent was obtained from all subjects involved in the study. The consent was obtained via the cover letter of both the electronic and paper survey, which participants reviewed prior to starting the survey. The consent form clarified the voluntary nature of participation, the length of time required, and the purpose of the study. The form did not explicitly mention future data analyses. All responses were deidentified prior to analysis. Survey data were stored on a secure institutional cloud drive accessible only to members of the research team. Incentives for participation included the option to enter a raffle to win one of five US $50 gift cards. No identification of individual participants has been included in this manuscript or supplementary materials.

## Results

The study identified differences in respondents’ demographic characteristics based on the survey completion mode. In phase 1, 224 electronic surveys were completed. In phase 1, 359 surveys were completed, of which 124 were electronic and 235 were paper surveys. Table 1 presents the sociodemographic characteristics of participants from phase 1 and phase 2.

**Table 1.** Sociodemographic characteristics of survey respondents (phase 1 and phase 2)^a^.

Characteristic	Phase 1^b^ electronic (n=224), n (%)	Phase 2^c^ electronic (n=124), n (%)	Phase 2 paper (n=235), n (%)
Gender			
Woman	125 (55.8)	53 (42.7)	149 (63.4)
Man	94 (42)	62 (50)	81 (34.5)
Other	2 (0.9)	2 (1.6)	2 (0.9)
Prefer not to answer	3 (1.3)	7 (5.65)	3 (1.28)
Sexual orientation			
Heterosexual	142 (67)	104 (83.9)	150 (63.8)
Homosexual	39 (18.4)	8 (6.5)	11 (4.7)
Other	31 (14.6)	3 (2.4)	21 (8.9)
Prefer not to answer	12 (5.4)	9 (7.3)	52 (22.1)
Age (y)			
18‐24	23 (10.3)	20 (16.1)	4 (1.7)
25‐34	40 (17.9)	43 (34.7)	9 (3.8)
35‐44	36 (16)	15 (12.1)	22 (9.4)
45‐54	36 (16)	10 (8.1)	30 (12.8)
55‐64	35 (15.6)	19 (15.3)	35 (14.9)
65‐74	34 (15.2)	8 (6.5)	65 (27.7)
≥75	8 (3.6)	2 (1.6)	23 (9.8)
Prefer not to answer	12 (5.4)	0 (0)	0 (0)
Preferred language for survey			
English	167 (74.6)	100 (80.7)	12 (5.1)
Vietnamese	57 (25.4)	24 (19.4)	223 (94.9)
Insurance status			
Insured	194 (86.6)	106 (85.5)	205 (87.2)
Uninsured	23 (10.3)	9 (7.3)	21 (8.9)
Prefer not to answer	7 (3.1)	9 (7.3)	9 (3.8)
Education			
Less than high school	25 (11.2)	12 (9.7)	69 (29.4)
High school or GED^d^ equivalent	47 (21.0)	21 (16.9)	76 (32.3)
Some college	37 (16.5)	25 (20.2)	20 (8.5)
Associate or college degree	87 (38.8)	46 (37.1)	48 (20.4)
Professional or doctorate degree	21 (9.4)	18 (14.5)	4 (1.7)
Prefer not to answer	7 (3.1)	8 (6.5)	18 (7.7)
COVID-19 vaccination status			
Fully vaccinated	181 (80.8)	102 (82.3)	206 (87.7)
First dose of a 2-dose vaccine	20 (8.9)	14 (11.3)	17 (7.2)
Not vaccinated	9 (4.0)	3 (2.4)	8 (3.4)
Prefer not to answer	14 (6.3)	5 (4)	4 (1.70)
Need assistance reading written health information	34 (15.18)	39 (31.5)	165 (70.2)

a Descriptive characteristics of Vietnamese American adults in Texas who participated in a cross-sectional COVID-19 study conducted between 2021 and 2023.

b Phase 1 (September 20, 2021-March 4, 2022) involved online-only surveys.

c Phase 2 (December 26, 2022-April 30, 2023) involved both online and paper surveys*.*

d GED: General Education Development.

Of the 224 phase 1 respondents, 125 (55.8%) identified as female, 94 (42%) as male, and 2 (0.9%) as other genders. Regarding language preference, 167 (74.6%) participants completed the survey in English, while 57 (25.4%) completed it in Vietnamese. In phase 1, 194 (86.6%) participants were insured, while 23 (10.3%) were uninsured. Educational attainment was nearly evenly distributed, with 108 (48.2%) participants reporting a college degree or higher and 109 (48.7%) reporting less than a college degree. In this phase, 181 (80.8%) participants were fully vaccinated, 20 (8.9%) received the first dose of a 2-dose vaccine, and 9 (4%) were unvaccinated.

In phase 2, which incorporated both electronic (n=124, 34.5%) and paper (n=235, 65.5%) surveys, 359 participants completed the survey. Of these, 202 (56.3%) identified as female participants, 143 (39.8%) as male participants, and 4 (1.1%) as other genders. Seven (1.9%) preferred not to answer. In terms of language preference, 112 (31.2%) participants completed the survey in English, while 247 (68.8%) completed it in Vietnamese. Among the respondents, 311 (86.6%) were insured, 30 (8.4%) were uninsured, and 18 (5%) preferred not to answer. Educational attainment was reported as 98 (27.3%) with a college degree or higher, 235 (65.5%) with less than a college degree, and 26 (7.2%) preferred not to answer. In terms of vaccination status, 308 (85.8%) were fully vaccinated, 31 (8.6%) had received the first dose of a 2-dose vaccine, 11 (3.1%) were unvaccinated, and 9 (2.5%) preferred not to answer. Further details on the breakdown between electronic and paper survey responses are presented in Table 1.

Table 2 highlights the multivariate logistic regression analysis using data gathered solely within phase 2. Being a Vietnamese speaker was the strongest predictor of completing a paper survey (AOR 100.9, 95% CI 24.3‐418.9; *P*<.001). Additionally, female participants were significantly more likely to complete a paper survey than male participants (AOR 5.09, 95% CI 1.43‐18.1; *P*=.01). Conversely, respondents with a history of COVID-19 infection (AOR 0.16, 95% CI 0.05‐0.52; *P*=.002), those with higher education levels (AOR 0.18, 95% CI 0.05‐0.67; *P*=.01), and those who expressed a strong willingness to participate in COVID-19 clinical trials (AOR 0.21, 95% CI 0.06‐0.81; *P*=.02) were more likely to complete the survey electronically.

**Table 2.** Multivariate logistic regression examining differences in characteristics between participants who completed paper versus electronic surveys in phase 2. Results from a multivariate logistic regression analysis examining associations between participant sociodemographic characteristics and survey modality among Vietnamese American adults in Texas during phase 2 (December 26, 2022-April 30, 2023) of a cross-sectional COVID-19 study*.* Measures the odds of a respondent having completed a paper survey in the survey mode of administration versus an electronic survey.

Sociodemographic variables	AOR^g^ (95% CI)	*P* value
Language use (reference: English)		
Vietnamese	100.91 (24.31‐418.95)	<.001
Age		
Per increasing year	1.03 (0.99‐1.07)	.11
Sex (reference: biological male)		
Biological female	5.09 (1.43‐18.10)	.01
Health insurance status (reference: uninsured)		
Insured	0.33 (0.04‐3.06)	.33
Educational attainment (reference: less than high school)		
Some college	0.23 (0.05‐1.12)	.07
College or higher	0.18 (0.05‐0.67)	.01
History of prior COVID-19 infection (reference: no)		
Yes	0.16 (0.05‐0.52)	.002
Completed COVID-19 vaccination^b^ (reference: no)		
Yes	1.45 (0.23‐9.10)	.69
Great deal of trust in the following sources^c^ (reference: less than a great deal of trust)		
NIH^d^	0.57 (0.12‐2.75)	.48
Health care provider	3.63 (0.54‐24.59)	.19
Local community clinic	1.28 (0.19‐8.78)	.80
University hospital	1.68 (0.24‐11.73)	.60
Pharmaceutical company	1.47 (0.20‐10.92)	.71
Researchers	0.81 (0.19‐3.49)	.78
Friend/family	0.35 (0.04‐3.11)	.35
FDA^e^	1.11 (0.33‐3.79)	.86
Level of willingness to participate in COVID-19 clinical trial (reference: less than high willingness^f^)		
High willingness^f^	0.21 (0.06‐0.81)	.02

a AOR: adjusted odds ratio.

b Completed COVID-19 vaccination series (either 1 vaccination of a 1-vaccination series or both vaccinations of a 2-vaccination series).

c Defined as the proportion of those who trust each row’s source a great deal versus those who endorse less than a great deal of trust.

d NIH: National Institutes of Health.

e FDA: Food and Drug Administration.

f Defined as a high level of willingness to participate in a COVID-19 clinical trial (6 or 7 on the Likert scale) versus those with less than a high level of willingness (≤5 on the 7-point Likert scale).

## Discussion

### Principal Findings

This study provides insights into the challenges of conducting public health research among Vietnamese Americans, a historically underrepresented population in public health data. The initial reliance on electronic surveys in phase 1 predominantly engaged younger, more educated, and technologically proficient individuals, leading to the unintended exclusion of key population segments, including older adults, non-English speakers, and those with lower educational attainment [38]. These social determinants are well-recognized factors that contribute to health disparities, not only in terms of access to health care but also in the context of research participation and representation [1,8,40,41]. Importantly, survey completion in a given modality reflects both access to that modality and willingness to engage with it. Digital participation requires digital access, just as paper-based participation presupposes in-person contact. Therefore, differences in survey completion across modalities should be interpreted not only as participant preference, but as an outcome shaped by where and how participants encountered the survey.

Demographic differences between respondents who completed paper versus electronic surveys highlight the limitations of digital-only data collection. Studies conducted among ethnic subpopulations have examined response rates from web or mail surveys [42]. However, this study is one of the first to highlight this in a population where digital literacy is a barrier. For instance, research involving women with cancer found that in ethnic subpopulation questionnaires, data quality and responses significantly varied by mode of survey administration [43]. These findings align with the results of our study, reinforcing the value of accessible survey methodologies that account for the diverse needs of marginalized populations.

### Nonmedical Drivers of Health

#### Language and Digital Access Barriers

Language accessibility is an important nonmedical driver of health that not only influences access to health care information and services but also impacts participation in health research, contributing to health disparities [44,45]. Research studies conducted exclusively in English may systematically exclude non-English speakers, limiting the representation and generalizability of findings. A systematic review assessing health outcomes in Asian American subgroups in the United States found that all 76 studies only considered English-speaking individuals, underscoring barriers faced by linguistically diverse populations in research participation [1]. In response, this study partnered with CBOs in a CEnR approach to translate surveys into Vietnamese, improving accessibility for a historically underrepresented subgroup and facilitating engagement in a culturally relevant manner [46]. This study’s findings indicate that Vietnamese language preference was significantly associated with completing paper surveys compared to electronic surveys. While online surveys are commonly used in large-scale health studies due to their convenience, cost-effectiveness, and onsite data error prevention, they may unintentionally exclude individuals with limited internet access or digital literacy skills [47]. In phase 2 of our study, 90.3% (223/247) of Vietnamese-speaking participants completed paper surveys compared to 10.7% (12/112) of English-speaking participants, suggesting that an electronic-only approach may underrepresent certain populations. Paper surveys are often more accessible to individuals who lack reliable internet access or familiarity with digital platforms, which may be particularly relevant for older Vietnamese Americans, who may encounter linguistic barriers and have lower levels of digital literacy than younger individuals [6,48,49]. Additionally, a potential contributing factor may be the recruitment context. Given that in phase 2, paper surveys were distributed in part at community events via CBOs (eg, BPSOS), whose mission was to support recent Vietnamese immigrants, refugees, and low-income families, participants may have felt more comfortable participating in research through organizations they trust, which could partly explain the higher rates of paper survey completion among Vietnamese-speaking respondents. Overall, a multipronged recruitment strategy, including both translated paper and electronic surveys, may include a broader range of participants and mitigate research participation disparities among linguistically diverse communities.

#### Education and Digital Disparities

One limitation of phase 1 was the underrepresentation of participants with less than a high school education. The 2022 Asian Americans, Native Hawaiians, and Pacific Islanders (AANHPI) report used data from the 2020 American Community Survey, applying a rolling multiyear sampling methodology to generate weighted socioeconomic and demographic characteristics estimates [38,50,51]. While the 2022 AANHPI report found that 25% of Vietnamese Americans had not completed high school, only 11.2% (25/224) of our phase 1 respondents fell into this category [38,49,50]. However, in phase 2, this percentage increased to 22.6% (69/305), suggesting that paper-based survey administration may improve participation among individuals with lower educational attainment. This finding aligns with prior research highlighting that education level is a determinant of digital literacy and online survey participation [9,51]. Consequently, digital-only survey methods may inadvertently exclude this population.

#### Health Insurance

Comparing our sample demographics to the 2022 AANHPI report, we found similar rates of uninsured participants in both study phases (phase 1: 10.3%, 23/224 vs phase 2: 8.4%, 30/359) [50,51]. Insurance status often correlates with socioeconomic factors influencing digital access, but our findings suggest that insurance coverage alone did not significantly predict survey modality preference. Uninsured participants were neither disproportionately excluded from digital surveys nor more likely to complete paper surveys, indicating that other social determinants, such as language, education, or gender, played a larger role in survey participation.

### Role of Community-Based Organizations in Community-Engaged Research

Given the digital and language barriers identified in our study, CBOs played a critical role in bridging accessibility gaps, improving representation, recruiting non-English speakers, distributing paper surveys, and increasing research trust. Often, CBO’s deep-rooted connections within the community and understanding of cultural and linguistic nuances uniquely position them to enhance research participation [32,34,36].

CBO involvement can ensure that research is methodologically sound, culturally appropriate, and respectful of the community’s unique needs [33-36]. Prior studies have demonstrated that CBOs can effectively bridge the gap between researchers and participants, fostering trust and facilitating engagement in a way that traditional research institutions often cannot [35,52]. This study adds to the growing body of evidence supporting the involvement of CBOs, demonstrating that their contributions are critical for recruitment and ensuring that research methodologies align with the target population’s cultural and linguistic needs [35].

### Limitations

This study contains some limitations. Using a cross-sectional survey limits the ability to establish causality, preventing generalizability beyond the sample population. In addition, this research was conducted within a specific geographic and demographic context, focusing exclusively on Vietnamese Americans in Texas. As such, the findings may not fully reflect the experiences of Vietnamese Americans in other regions or contexts. Additionally, this survey instrument did not assess participants’ years of immigration to the United States, which may influence acculturation, language proficiency, and technology use [8]. Indeed, neither sample in this study (electronic or paper survey respondents) can be assumed to reflect or represent the broader population of Vietnamese Americans, even in the region studied here. Because recruitment was based on convenience sampling through CBOs, events, and listservs, the sample is subject to selection bias. In-person recruitment using paper surveys took place, in part, at Houston-area events offering free health care screenings. This likely attracts a different population than who receives a recruitment email from a Vietnamese American health professional listserv. In addition, participants who are more health-seeking or community-engaged may be overrepresented across respondents. The differences between the two groups of respondents even suggest that there is heterogeneity in how such a representative sample might be constructed but do not suggest that one or the other is more generalizable. In addition, overall, the sample size collected for either group is small, and the multivariate approach risks overfitting the models reported here, resulting in large (occasionally very large) CIs. As a result, point estimates of effect sizes should be treated as exploratory findings, and the risk of type II error (falsely null associations) is higher than the usual likelihood.

Although our sample size in our exploratory cross-sectional study design limits our external validity of our exploratory observations to the broader Vietnamese American population, statistical methods such as poststratification or propensity score adjustment would enable future researchers to mitigate selection bias by reweighting the analytic sample to reflect the distribution of observed covariates in the general population.

### Conclusion

In conclusion, this study underscores the importance of using flexible and adaptive research methodologies when engaging with marginalized populations. The combination of digital and paper survey methods, informed by community insights and tailored to the specific needs of the Vietnamese American community, proved to be an effective strategy for enhancing participation and representativeness. These findings have broader implications for public health research, particularly in addressing health disparities among historically marginalized populations to achieve health equity. As the field moves toward more inclusive and equitable research practices, researchers must continue to draw on the expertise of community partners and pay close attention to the social determinants of health that shape participation and outcomes. By doing so, public health research can better serve the needs of all populations, ultimately contributing to reducing health disparities and promoting health equity.

In summary, this study underscores the role of CBOs and CEnR in enhancing the inclusivity and representativeness of public health research. The study achieved a more comprehensive representation of the Vietnamese American community by integrating paper surveys and bilingual recruitment strategies. Future public health research should continue prioritizing the involvement of CBOs and CEnR to ensure that different population segments are adequately represented. This approach is fundamental to addressing health disparities and developing public health interventions responsive to different communities’ needs.

## Supplementary material

10.2196/77520 Multimedia Appendix 1 Common survey (English).

10.2196/77520 Multimedia Appendix 2 Common survey (Vietnamese).

## Abbreviations

AANHPI: Asian Americans, Native Hawaiians, and Pacific Islanders
AOR: adjusted odds ratio
BPSOS: Boat People SOS—Houston
CBO: community-based organization
CEAL: Community Engagement Alliance
CEnR: community-engaged research
NIH: National Institutes of Health
