# Supplementary material for: Survey Modalities and COVID-19 Vaccine Uptake in Vietnamese Americans: Cross-Sectional Study
Source: JMIR Public Health Surveill. 2026 Feb 25;12:e77520. doi: 10.2196/77520 (PMC12935459; doi:10.2196/77520)
Supplement: Multimedia Appendix 1 [file publichealth-v12-e77520-s001.pdf]

CEAL Common Survey 2 Tier 1

1. About how long has it been since you last saw a doctor or other health care professional about your health?

- ☐ Never
- ☐ Within the past 12 months/1 year
- ☐ 1 to 2 years ago
- ☐ 3 to 4 years ago
- ☐ 5 to 9 years ago
- ☐ 10 years ago, or more
- ☐ Prefer not to answer

2. Do you have any kind of health insurance or health care plan

- ☐ Yes → [IF YES]
- ☐ No → [IF NO]
- ☐ Don't Know
- ☐ Prefer not to answer

[IF NO] Did you lose health coverage during the COVID-19 pandemic?

- ☐ Yes
- ☐ No
- ☐ Don't Know
- ☐ Prefer not to answer

[IF YES] What is the primary kind of health insurance or health care plan that you have now?

- ☐ Private health insurance through a job or school
- ☐ Insurance bought through a government exchange such as healthcare.gov
- ☐ Insurance bought from a health plan or company
- ☐ Medicare
- ☐ Medi-Gap
- ☐ Medicaid
- ☐ CHIP or kid’s state insurance
- ☐ Military health care
- ☐ Indian Health Service
- ☐ Other: ___________
- ☐ Don’t Know
- ☐ Prefer not to answer

3. The COVID-19 pandemic may cause challenges for some people, whether they get COVID-19 or not. In the past month, have you experienced any of the below challenges?

	No, this is not a challenge	Yes, this is a minor challenge	Yes, this is a major challenge	Prefer not to answer
**A. Getting the health care I need (including mental health)**	☐	☐	☐	☐
**B. Having a place to live**	☐	☐	☐	☐
**C. Getting enough food to eat**	☐	☐	☐	☐
**D. Having clean water to drink**	☐	☐	☐	☐
**E. Getting the medications I need**	☐	☐	☐	☐
**F. Getting where I need to go**	☐	☐	☐	☐
**G. Taking care of my children or other people in my care**	☐	☐	☐	☐

4. How much do you trust each of these sources to provide correct information about COVID-19?

	Not at all	A little	A great deal	Don't know	Does not apply	Prefer not to answer
A. Your doctor or healthcare provider	☐	☐	☐	☐	☐	☐
B. Your faith leader (e.g., priest, minister, monk)	☐	☐	☐	☐	☐	☐
C. People you go to work or class with or other people you know	☐	☐	☐	☐	☐	☐
D. News on the radio, TV, online, or in newspapers	☐	☐	☐	☐	☐	☐
E. Your contacts on social media	☐	☐	☐	☐	☐	☐
F. Federal government	☐	☐	☐	☐	☐	☐
G. State and/or local government	☐	☐	☐	☐	☐	☐
H. Tribal leadership	☐	☐	☐	☐	☐	☐
I. The Centers for Disease Control and Prevention (CDC)	☐	☐	☐	☐	☐	☐
J. A community organization that provides services and assistance where you live	☐	☐	☐	☐	☐	☐

5. How much do you trust the United States Food and Drug Administration (FDA) to ensure the COVID-19 vaccine is safe for the public?

☐ Fully trust
☐ Mostly trust
☐ Somewhat trust
☐ Do not trust
☐ Prefer not to answer

6. How much do you trust the federal government to ensure a COVID-19 vaccine is safe for children?

☐ Fully trust
☐ Mostly trust
☐ Somewhat trust
☐ Do not trust
☐ Prefer not to answer

7. Have you ever been tested for COVID-19?

☐ Yes →
☐ No
☐ Prefer not to answer

[IF YES] How many times did you get the COVID-19 test?

☐ One
☐ Two
☐ Three
☐ Four or more
☐ Prefer not to answer

[IF YES] Have you ever tested positive for COVID-19?

☐ Yes
☐ No
☐ Don't Know
☐ Prefer not to answer

8. Have you received at least one dose of the COVID-19 vaccine?

- ☐ Yes, got one-dose vaccine →
- ☐ Yes, got first dose of two-dose vaccine →
- ☐ Yes, got both doses of two-dose vaccine →
- ☐ No, have not gotten the vaccine ↓
- ☐ Don't know
- ☐ Prefer not to answer

8A. [IF NO] How likely are you to get a COVID-19 vaccine in the next 3 months?

1	2	3	4	5	6	7
○	○	○	○	○	○	○
Not likely at all						Extremely likely

☐ Prefer not to

8B. [IF NO] What are the reasons why you have not yet gotten a COVID-19 vaccine? (Select all that apply.)

- ☐ I don't like needles.
- ☐ I don't think I am at risk.
- ☐ I don't think vaccines work very well.
- ☐ I don't trust that the vaccine will be safe.
- ☐ I'm concerned about side effects from the vaccine.
- ☐ People in my family or community do not approve of the vaccine.
- ☐ I already had COVID-19 so I do not think I need to get the vaccine.
- ☐ It conflicts with my religious beliefs.
- ☐ I am worried about being asked to show my ID at a vaccine appointment.
- ☐ I am worried about being infected with COVID-19 by going to a vaccination location.
- ☐ Other: ________________________________
- ☐ Prefer not to answer

8C. [IF NO] What has made it hard for you to get a COVID-19 vaccine? (Select all that apply.)

- ☐ I can't pay for it.
- ☐ I don't know where to get vaccinated.
- ☐ I don't have transportation to get there.
- ☐ I cannot take time off work to get the vaccine.
- ☐ I don't know how to make an appointment for a vaccination.
- ☐ I don't have someone to watch my children/other people in my care while I go.
- ☐ They don't speak my language at the vaccination location.
- ☐ I don't have a social security number or government issued ID to get the COVID-19 vaccine.
- ☐ Other: ____
- ☐ Prefer not to answer

8D. [IF YES] Was there anything that made it hard to get a COVID-19 vaccine? (Please choose all that apply)

- ☐ I didn't know how to get an appointment for my vaccination.
- ☐ The appointment took too long.
- ☐ I was worried about being asked to show my ID at a vaccine appointment.
- ☐ I didn't have transportation to or from a vaccination location.
- ☐ Vaccination locations were too far or hard to get to.
- ☐ I didn't know where to go for my vaccination.
- ☐ I didn't have someone to watch my children/other people in my care while I went.
- ☐ I couldn't take time off work for my vaccination.
- ☐ They didn't speak my language at the vaccination location.
- ☐ I was not able to access information about the COVID-19 vaccine in my preferred language.
- ☐ I don't trust that the vaccine will be safe.
- ☐ I was worried about being infected with COVID-19 by going to a vaccination location.
- ☐ I was concerned about side effects from the vaccine.
- ☐ I don't think vaccines work very well.
- ☐ I'm allergic to vaccines.
- ☐ I don't like needles.
- ☐ It conflicts with my religious beliefs.
- ☐ People important to me did not approve of me getting the vaccine.

9. Why would you (or did you) get a COVID-19 vaccine? Select all that apply.

- ☐ I want to keep my family safe.
- ☐ I want to keep my community safe.
- ☐ I want to keep myself safe.
- ☐ I have a chronic health problem, like asthma or diabetes.
- ☐ My doctor told me to get a COVID-19 vaccine.
- ☐ I don't want to get seriously sick from COVID-19.
- ☐ I want to feel safe around other people.
- ☐ I believe life won't go back to normal until most people get a COVID-19 vaccine.
- ☐ My community or family expects me to.
- ☐ I would not get a COVID-19 vaccine.
- ☐ Other: _________________
- ☐ Prefer not to answer

10. How confident are you that the COVID-19 vaccines currently available in the U.S. are safe?

- ☐ Very confident
- ☐ Somewhat confident
- ☐ Not too confident
- ☐ Not at all confident
- ☐ Don't know
- ☐ Prefer not to answer

11. Have you ever enrolled in a COVID-19 clinical trial?

- ☐ Yes, I signed up for a clinical trial for a COVID-19 vaccine.
- ☐ Yes, I signed up for a clinical trial for a COVID-19 treatment.
- ☐ No, I have never signed up for a COVID-19 clinical trial.
- ☐ Prefer not to answer

12. Below are sources of information about COVID-19 clinical trials. How much do you trust each of these sources to give correct information? (Select one response for each.)

	A great deal	A fair amount	Not very much	Not at all	No opinion	Prefer not to answer
**A. The National Institutes of Health (NIH)**	☐	☐	☐	☐	☐	☐
**B. Your doctor or healthcare provider**	☐	☐	☐	☐	☐	☐
**C. Your local health care clinic or hospital**	☐	☐	☐	☐	☐	☐
**D. University hospitals**	☐	☐	☐	☐	☐	☐
**E. Companies that make drugs for medical use**	☐	☐	☐	☐	☐	☐
**F. People who do research**	☐	☐	☐	☐	☐	☐
**G. Friends, family, and community leaders**	☐	☐	☐	☐	☐	☐

13. If you get COVID-19, how willing would you be to sign up for a clinical trial for a COVID-19 treatment?

1	2	3	4	5	6	7	☐
Not at all willing						Very willing	Prefer not to answer

14. What month and year were you born?

☐ __ __/ __ __ __ __
M M Y Y Y Y

☐ Prefer not to answer

15. What is your gender?

☐ Man
☐ Woman
☐ Transgender Female or Trans Woman
☐ Transgender Male or Trans Male
☐ Nonbinary, Genderqueer, or Genderfluid
☐ I would describe my gender as: ________________
☐ Prefer not to answer

16. Which of the following best describes how you think of yourself?

☐ Gay
☐ Lesbian
☐ Straight (that is, not gay, lesbian, or bisexual)
☐ Bisexual
☐ Other
☐ Prefer not to answer

17. Are you of Hispanic or Latino origin?

☐ No
☐ Yes →
☐ Prefer not to answer

[IF YES] Which of the following best describes your Hispanic/Latino heritage?

☐ Cuban
☐ Colombian
☐ Dominican
☐ Guatemalan
☐ Honduran
☐ Mexican or Mexican American or Chicano
☐ Puerto Rican
☐ Salvadoran
☐ Specify: (for example, Ecuadorian, Nicaraguan, Peruvian, Spaniard, Venezuelan)
____
☐ Prefer not to answer

18. Which of the following best describes your race? (Select all that apply.)

- ☐ American Indian or Alaska Native
  - ☐ Enrolled in a federally recognized tribe (Specify: _______________)
  - ☐ Enrolled in a state recognized tribe (Specify: ____________________)
  - ☐ Eligible for enrollment, but I am not enrolled in my tribe (Specify: _____________)
  - ☐ Not enrolled, but I am a descendent of an American Indian or Alaska Native tribe
  - ☐ Not applicable, my Indigenous People/Community do not have tribal enrollment
  - ☐ Other answer not specified here (Specify: _____________________)

  Is your location:
    - ☐ Urban
    - ☐ Rural
    - ☐ On a Reservation
    - ☐ Other (Please specify):____________
- ☐ Asian
  - ☐ Asian Indian
  - ☐ Chinese
  - ☐ Filipino
  - ☐ Japanese
  - ☐ Korean
  - ☐ Pakistani
  - ☐ Thai
  - ☐ Vietnamese
  - ☐ Cambodian
  - ☐ Hmong
  - ☐ Specify: (for example, Bangladeshi, Bhutanese, Burmese, Indonesian, Laotian, Malaysian, Mongolian, Nepalese, Okinawan, Sri Lankan, Taiwanese) ___________________________
- ☐ Black or African American
  - ☐ African American
  - ☐ Ethiopian
  - ☐ Haitian
  - ☐ Jamaican
  - ☐ Nigerian
  - ☐ Somalian
  - ☐ Ghanaian
  - ☐ Trinidadian and Tobagonian
  - ☐ Specify: (for example, Barbadian, Cape Verdean, South African) ____________
- ☐ Native Hawaiian/Pacific Islander
  - ☐ Chamorro
  - ☐ Fijian
  - ☐ Marshallese
  - ☐ Native Hawaiian
  - ☐ Palauan/Belauan
  - ☐ Samoan
  - ☐ Tongan
  - ☐ Chuukese
  - ☐ Pohnpeian
  - ☐ Specify: (for example, Carolinian, I-Kiribati, Kosraean, Mariana Islander, Papua New Guinean, Saipanese, Tahitian, Tokelauan, Yapese) _______________________
- ☐ White
  - ☐ English
  - ☐ French
  - ☐ German
  - ☐ Irish
  - ☐ Italian
  - ☐ Polish
  - ☐ Specify: (for example, Scottish, Norwegian, Dutch) _______________________
- ☐ Prefer not to answer

19. What is the highest degree or level of school you have completed?

- ☐ Some elementary school (Grades 1-5)
- ☐ Elementary school graduate (Grades 1-5)
- ☐ Some middle school (Grades 6-8)
- ☐ Middle school graduate (Grades 6-8)
- ☐ Some high school (Grades 9-12)
- ☐ High school graduate (Grades 9-12)
- ☐ GED
- ☐ Some college – no degree
- ☐ Associate's or technical degree (for example, AA or AS)
- ☐ Bachelor's degree (for example, BA, BS, or AB)
- ☐ Graduate degree (for example, MA or PhD)
- ☐ Prefer not to answer

20. How many people live in your household right now? Include yourself, any other adults, and any children.

___ Adults (age 18 or older) and ___ Minors (younger than age 18)

- ☐ Prefer not to answer

21. Which best describes your current employment status (select all that apply)?

- ☐ Working for pay—part time (less than 40 hours a week)
- ☐ Working for pay—full time (40 hours a week or more)
- ☐ Working without pay (for example, as an intern)
- ☐ On leave from work
- ☐ Unemployed and looking for a job
- ☐ Unemployed and NOT looking for a job
- ☐ Retired from work
- ☐ Staying at home, taking care of the home or of others
- ☐ Not able to work because of a disability
- ☐ Going to school
- ☐ Other: ______
- ☐ Prefer not to answer

22. Do you speak a language other than English at home?

- ☐ Yes → **[IF YES]** What language(s)?___________________________
- ☐ No
- ☐ Prefer not to answer

23. How often do you need someone to help you read written information from your doctor or drug store?

- ☐ Never
- ☐ Rarely
- ☐ Sometimes
- ☐ Often
- ☐ Always
- ☐ Prefer not to answer

End of Survey.

Thank you! If you would like to enter a raffle for a $50 gift card, please fill out the next page

Q24: If you would like to be entered into a raffle for one of five $50 Visa gift cards, please provide your first name, last name, email address or phone number.

Name ___________________________________ ___________________________________

Print First Name *Print Last Name*

____ __ __ __- __ __ __- __ __ __ __

Email address *Phone number*
