# Supplementary material for: Survey Modalities and COVID-19 Vaccine Uptake in Vietnamese Americans: Cross-Sectional Study
Source: JMIR Public Health Surveill. 2026 Feb 25;12:e77520. doi: 10.2196/77520 (PMC12935459; doi:10.2196/77520)
Supplement: Multimedia Appendix 2 [file publichealth-v12-e77520-s002.pdf]

CEAL Khảo Sát 2 Bậc 1

1. Đã bao lâu rồi kể từ lần gần nhất quý vị gặp một bác sĩ hoặc người có chuyên môn chăm sóc sức khỏe về sức khỏe của quý vị?

- ☐ Chưa bao giờ
- ☐ Trong vòng 12 tháng/1 năm qua
- ☐ 1 đến 2 năm trước
- ☐ 3 đến 4 năm trước
- ☐ 5 đến 9 năm trước
- ☐ 10 năm trước hoặc hơn
- ☐ Không muốn trả lời

2. Quý vị có bất kỳ loại bảo hiểm y tế hoặc chương trình chăm sóc sức khỏe nào không?

- ☐ Có →
- ☐ Không →
- ☐ Không biết
- ☐ Không muốn trả lời

[NẾU KHÔNG] Quý vị có bị mất bảo hiểm y tế trong thời gian xảy ra đại dịch COVID-19 không?

- ☐ Có
- ☐ Không
- ☐ Không biết
- ☐ Không muốn trả lời

[NẾU CÓ] Quý vị hiện có loại bảo hiểm y tế hay chương trình chăm sóc sức khỏe chính nào?

- ☐ Bảo hiểm y tế tư nhân thông qua công việc hoặc trường học
- ☐ Bảo hiểm mua qua sàn giao dịch của chính phủ như healthcare.gov
- ☐ Bảo hiểm mua từ chương trình chăm sóc sức khỏe hoặc công ty
- ☐ Medicare
- ☐ Medi-Gap
- ☐ Medicaid
- ☐ CHIP hoặc bảo hiểm của tiểu bang cho trẻ em
- ☐ Chương trình chăm sóc sức khỏe của quân đội
- ☐ Dịch vụ chăm sóc sức khỏe cho người da đỏ
- ☐ Khác: ____________
- ☐ Không biết
- ☐ Không muốn trả lời

3. Đại dịch COVID-19 có thể gây ra khó khăn cho một số người, dù họ có mắc COVID-19 hay không. Trong tháng qua, quý vị có gặp bất kỳ khó khăn nào dưới đây không?

	Không, đây không phải là khó khăn	Có, đây là khó khăn nhỏ	Có, đây là khó khăn lớn	Không muốn trả lời
**A. Tìm dịch vụ chăm sóc sức khỏe tôi cần (kể cả sức khỏe tâm thần)**	☐	☐	☐	☐
**B. Có nơi ở**	☐	☐	☐	☐
**C. Có đủ thực phẩm**	☐	☐	☐	☐
**D. Có nước sạch để uống**	☐	☐	☐	☐
**E. Có loại thuốc tôi cần**	☐	☐	☐	☐
**F. Đến được nơi tôi cần tới**	☐	☐	☐	☐
**G. Chăm sóc cho con cái tôi hoặc những người khác thuộc trách nhiệm của tôi**	☐	☐	☐	☐

4. Quý vị tin tưởng về độ chính xác của các nguồn thông tin về COVID-19 sau đến mức nào?

	Không hề	Một chút	Nhiều	Không biết	Không áp dụng	Không muốn trả lời
A. Bác sĩ hoặc nhà cung cấp dịch vụ chăm sóc sức khỏe của quý vị	☐	☐	☐	☐	☐	☐
B. Lãnh đạo tôn giáo (ví dụ: thầy tu, mục sư, nhà sư)	☐	☐	☐	☐	☐	☐
C. Những người quý vị đi làm hoặc học cùng hoặc những người khác quý vị biết	☐	☐	☐	☐	☐	☐
D. Tin tức trên đài phát thanh, TV, mạng hoặc báo	☐	☐	☐	☐	☐	☐
E. Người quen trên mạng xã hội trên mạng xã hội	☐	☐	☐	☐	☐	☐
F. Chính phủ liên bang	☐	☐	☐	☐	☐	☐
G. Chính quyền tiểu bang và/hoặc địa phương	☐	☐	☐	☐	☐	☐
H. Lãnh đạo bộ lạc	☐	☐	☐	☐	☐	☐
I. Trung Tâm Phòng Ngừa và Kiểm Soát Dịch Bệnh (CDC)	☐	☐	☐	☐	☐	☐
J. Một tổ chức cộng đồng cung cấp dịch vụ và hỗ trợ ở nơi quý vị sinh sống	☐	☐	☐	☐	☐	☐

5. Quý vị tin tưởng Cục Quản Lý Thực phẩm và Dược phẩm Hoa Kỳ (FDA) trong việc bảo đảm vắc-xin COVID-19 an toàn cho công chúng đến mức nào?

- ☐ Hoàn toàn tin tưởng
- ☐ Hầu như tin tưởng
- ☐ Có chút tin tưởng
- ☐ Không tin tưởng
- ☐ Không muốn trả lời

6. Quý vị tin tưởng chính phủ liên bang đảm bảo vắc-xin COVID-19 an toàn cho trẻ em đến mức nào?

- ☐ Hoàn toàn tin tưởng
- ☐ Hầu như tin tưởng
- ☐ Có chút tin tưởng
- ☐ Không tin tương
- ☐ Không muốn trả lời

7. Quý vị đã xét nghiệm COVID-19 bao giờ chưa?

- ☐ Có →
- ☐ Không
- ☐ Không muốn trả lời

[NẾU CÓ] Quý vị đã xét nghiệm COVID-19 bao nhiêu lần rồi?

- ☐ Một
- ☐ Hai
- ☐ Ba
- ☐ Bốn hoặc hơn
- ☐ Không muốn trả lời

[NẾU CÓ] Quý vị đã có kết quả xét nghiệm dương tính với COVID-19 bao giờ chưa?

- ☐ Có
- ☐ Không
- ☐ Tôi không biết
- ☐ Không muốn trả lời

8. Quý vị đã tiêm ít nhất 1 liều vắc-xin COVID-19 chưa?

- ☐ Rồi, đã tiêm vắc-xin một liều →
- ☐ Rồi, đã tiêm mũi một của vắc-xin hai liều →
- ☐ Rồi, đã tiêm cả hai mũi của vắc-xin hai liều →
- ☐ Chưa, chưa tiêm vắc-xin ↓
- ☐ Không biết
- ☐ Không muốn trả lời

8A. [NẾU KHÔNG] Khả năng quý vị tiêm vắc-xin COVID-19 trong ba tháng tiếp theo là bao nhiêu?

1	2	3	4	5	6	7
○	○	○	○	○	○	○
Hoàn toàn không						Rất có thể

☐ Không muốn trả lời

8B. [NẾU KHÔNG] Đâu là lý do quý vị chưa tiêm vắc-xin COVID-19? (Chọn tất cả các mục phù hợp.)

- ☐ Tôi không thích kim tiêm.
- ☐ Tôi không nghĩ mình sẽ có nguy cơ.
- ☐ Tôi không nghĩ vắc-xin có hiệu quả tốt.
- ☐ Tôi không tin rằng vắc-xin sẽ an toàn.
- ☐ Tôi lo ngại về tác dụng phụ của vắc-xin.
- ☐ Người trong gia đình hoặc cộng đồng của tôi không ủng hộ vắc-xin.
- ☐ Tôi đã mắc COVID-19 nên tôi không nghĩ mình cần tiêm vắc-xin.
- ☐ Nó mâu thuẫn với tín ngưỡng tôn giáo của tôi.
- ☐ Tôi lo ngại sẽ bị yêu cầu trình ID khi tiêm vắc-xin.
- ☐ Tôi lo ngại bị nhiễm COVID-19 khi đi tiêm vắc-xin
- ☐ Khác ____________________________________
- ☐ Không muốn trả lời

8C. [NẾU KHÔNG] Điều gì khiến quý vị gặp khó khăn trong việc tiêm vắc-xin?
(Chọn tất cả các mục phù hợp.)

- ☐ Tôi không thể trả phí.
- ☐ Tôi không biết tiêm ở đâu.
- ☐ Tôi không có phương tiện để đến đó.
- ☐ Tôi không thể nghỉ làm để đi tiêm vắc-xin.
- ☐ Tôi không biết cách đặt lịch hẹn tiêm vắc-xin.
- ☐ Tôi không nhờ được ai trông con/người khác thuộc trách nhiệm của tôi để đi.
- ☐ Họ không nói ngôn ngữ của tôi tại điểm tiêm vắc-xin.
- ☐ Tôi không có số an sinh xã hội hay ID được chính quyền cấp để tiêm vắc-xin.
- ☐ Khác ____
- ☐ Không muốn trả

8D. [NẾU CÓ] Đã có bất kỳ điều gì khiến cho việc tiêm vắc-xin COVID-19 trở nên khó khăn không? (Vui lòng chọn tất cả các mục phù hợp)

- ☐ Tôi không biết cách đặt lịch hẹn tiêm vắc-xin.
- ☐ Buổi hẹn mất quá nhiều thời gian.
- ☐ Tôi lo ngại sẽ bị yêu cầu trình ID khi tiêm vắc-xin.
- ☐ Tôi không có phương tiện đến và đi từ điểm tiêm vắc-xin.
- ☐ Địa điểm tiêm vắc-xin quá xa hoặc khó đến.
- ☐ Tôi đã không biết đi đâu để tiêm vắc-xin.
- ☐ Tôi đã không nhờ được ai trông con/người khác thuộc trách nhiệm của tôi để đi.
- ☐ Tôi không thể nghỉ làm để đi tiêm vắc-xin.
- ☐ Họ không nói ngôn ngữ của tôi tại điểm tiêm vắc-xin.
- ☐ Tôi không thể truy cập thông tin về vắc-xin COVID-19 ở ngôn ngữ tôi mong muốn.
- ☐ Tôi không tin rằng vắc-xin sẽ an toàn.
- ☐ Tôi lo ngại bị nhiễm COVID-19 khi đi tiêm vắc-xin
- ☐ Tôi lo ngại về tác dụng phụ của vắc-xin.
- ☐ Tôi không nghĩ vắc-xin có hiệu quả tốt.
- ☐ Tôi bị dị ứng với vắc-xin.
- ☐ Tôi không thích kim tiêm.
- ☐ Nó mâu thuẫn với tín ngưỡng tôn giáo của tôi.
- ☐ Những người quan trọng với tôi đã không ủng hộ tôi tiêm vắc-xin.
- ☐ Không muốn trả lời

9. Tại sao quý vị lại tiêm vắc-xin? Chọn tất cả các mục phù hợp.

- ☐ Tôi muốn giữ an toàn cho gia đình tôi.
- ☐ Tôi muốn giữ an toàn cho cộng đồng của tôi.
- ☐ Tôi muốn giữ an toàn cho bản thân.
- ☐ Tôi bị bệnh mãn tính như hen suyễn hoặc tiểu đường.
- ☐ Bác sĩ của tôi khuyên tôi đi tiêm vắc-xin.
- ☐ Tôi không muốn bị bệnh COVID-19 nặng.
- ☐ Tôi muốn cảm thấy an toàn khi ở bên người khác.
- ☐ Tôi tin cuộc sống sẽ không trở lại bình thường cho tới khi hầu hết mọi người tiêm vắc-xin COVID-19.
- ☐ Cộng đồng và gia đình tôi muốn tôi làm như vậy.
- ☐ Tôi sẽ không tiêm vắc-xin COVID-19.
- ☐ Khác __________________
- ☐ Không muốn trả lời

10. Quý vị tưởng tin về độ an toàn của các vắc-xin COVID-19 hiện có tại Hoa Kỳ đến mức nào?

- ☐ Rất tin
- ☐ Có chút tin
- ☐ Không quá tự tin
- ☐ Không hề tự tin
- ☐ Không biết
- ☐ Không muốn trả lời

11. Quý vị có bao giờ đăng ký tham gia thử nghiệm lâm sàng COVID-19 chưa?

- ☐ Rồi, tôi ghi danh tham gia thử nghiệm lâm sàng của một vắc-xin COVID-19.
- ☐ Rồi, tôi ghi danh tham gia thử nghiệm lâm sàng của một phương pháp điều trị COVID-19.
- ☐ Chưa, tôi chưa bao giờ đăng ký tham gia thử nghiệm lâm sàng COVID-19.
- ☐ Không muốn trả lời

12. Dưới đây là các nguồn thông tin về thử nghiệm lâm sàng COVID-19. Quý vị tin tưởng đến mức nào về độ cung cấp thông tin chính xác của các nguồn thông tin sau? (Chọn một câu trả lời cho mỗi mục.)

	Nhiều	Tương đối	Không nhiều	Không hề	Không ý kiến	Không muốn trả lời
**A. Viện Y Tế Quốc Gia (NIH)**	☐	☐	☐	☐	☐	☐
**B. Bác sĩ hoặc nhà cung cấp dịch vụ chăm sóc sức khỏe của quý vị**	☐	☐	☐	☐	☐	☐
**C. Phòng khám hoặc bệnh viện chăm sóc sức khỏe tại địa phương**	☐	☐	☐	☐	☐	☐
**D. Các bệnh viện đại học**	☐	☐	☐	☐	☐	☐
**E. Các công ty sản xuất thuốc sử dụng cho y tế**	☐	☐	☐	☐	☐	☐
**F. Những người làm nghiên cứu**	☐	☐	☐	☐	☐	☐
**G. Bạn bè, gia đình và lãnh đạo cộng đồng**	☐	☐	☐	☐	☐	☐

13. Nếu quý vị mắc COVID-19, quý vị sẵn sàng như thế nào đối với việc đăng ký thử nghiệm lâm sàng một phương pháp điều trị COVID-19?

1	2	3	4	5	6	7	☐
○	○	○	○	○	○	○	Không muốn trả lời
Hoàn toàn không sẵn sàng						Rất sẵn sàng	

14. Quý vị sinh tháng nào và năm nào?

☐ __ __/ __ __ __ __
T T N N N N
☐ Không muốn trả lời

15. Giới tính của quý vị là gì?

☐ Nam
☐ Nữ
☐ Nữ chuyển giới
☐ Nam chuyển giới
☐ Phi nhị nguyên giới hay linh hoạt giới
☐ Giới tính của tôi là: ___________________________
☐ Không muốn trả lời

16. Quý vị tự nhận dạng mình như thế nào theo các lựa chọn dưới đây?

☐ Đồng tính nam
☐ Đồng tính nữ
☐ Chỉ thích người kháo giới (tức là không đồng tính nam, đồng tính nữ hay lưỡng tính)
☐ Lưỡng tính
☐ Khác
☐ Không muốn trả lời

17. Quý vị có nguồn gốc Tây Ban Nha hay Mỹ Latinh không?

☐ Không
☐ Có →
☐ Không muốn trả lời

[NẾU CÓ] Nếu có, điều nào sau đây mô tả nguồn gốc Tây Ban Nha/Mỹ Latinh của quý vị?

☐ Người Cuba
☐ Người Colombia
☐ Người Dominica
☐ Người Guatemala
☐ Người Honduras
☐ Người Mexico hay người Mỹ gốc Mexico hay người Chicano
☐ Người Puerto Rico
☐ Người El Salvador
☐ Nêu cụ thể: (Ví dụ, người Ecuador, người Nicaragua, người Peru, người Tây Ban Nha, người Venezuela) ________________________
☐ Không muốn trả lời

18. Điều nào sau đây mô tả chính xác chủng tộc của quý vị? (Chọn tất cả các mục phù hợp.)

- Điều nào sau đây mô tả chính xác chủng tộc của quý vị? (Chọn tất cả các mục phù hợp.)
  - Đã đăng ký vào một bộ tộc được liên bang công nhận (Nêu cụ thể ______)
  - Đã đăng ký vào một bộ tộc được tiểu bang công nhận (Nêu cụ thể: __________________)
  - Đủ điều kiện đăng ký, nhưng chưa đăng ký vào bộ tộc của tôi (Nếu cụ thể: _______________________)
  - Chưa đăng ký, nhưng tôi là hậu duệ của một bộ tộc người da đỏ châu Mỹ hoặc người Alaska bản địa
  - Không áp dụng, Nhóm Người/Cộng Đồng Người Bản Địa của tôi không có đăng ký bộ tộc
  - Câu trả lời khác chưa được nêu ở đây (Nêu cụ thể: __________________)

  Nơi ở của quý vị là:
    - Đô thị
    - Nông thôn
    - Ở vùng bảo tồn
    - Khác (Vui lòng nêu cụ thể):______
- Người châu Á
  - Người Ấn Độ
  - Người Trung Quốc
  - Người Philippines
  - Người Nhật Bản
  - Người Hàn Quốc
  - Người Pakistan
  - Người Thái Lan
  - Người Việt Nam
  - Người Cambodia
  - Người Hmong
  - Nêu cụ thể: (ví dụ: Người Bangladesh, Bhutan, Miến Điện, Indonesia, Lào, Mã Lai, Mông Cổ, Nepal, Nhật Okinawa, Sri Lanka, Đài Loan) ____________________________
- Người da đen hoặc người Mỹ gốc Phi
  - Người Mỹ gốc Phi
  - Người Ethiopia
  - Người Haiti
  - Người Jamaica
  - Người Nigeria
  - Người Somali
  - Người Ghana
  - Người Trinidad và Tobago
  - Nêu cụ thể: (ví dụ: Barbadian, Cape Verdean, South African) ____________________
- Người Hawaii bản địa/Người dân đảo Thái Bình Dương
  - Người Chamorro
  - Người Fiji
  - Người Marshall
  - Người Hawaii bản địa
  - Người Palau
  - Người Samoa
  - Người Tonga
  - Người Chuuk
  - Người Pohnpei
  - Nêu cụ thể: (ví dụ, người Caroline, Kiribati, Kosrae, quần đảo Mariana, Papua New Guinea, Saipan, Tahiti, Tokelau, Yap)______________
- Người da trắng
  - Người Anh
  - Người Pháp
  - Người Đức
  - Người Ireland
  - Người Italia
  - Người Ba Lan
  - Nêu cụ thể: (ví dụ, Người Scotland, Na-Uy, Hà Lan) _____________________
- Không muốn trả lời

19. Bằng hoặc cấp học cao nhất quý vị đã đạt được là gì?

- ☐ Một chút tiểu học (Lớp 1-5)
- ☐ Tốt nghiệp trường tiểu học (Lớp 1-5)
- ☐ Một trường trung học cơ sở nào đó (Lớp 6-8)
- ☐ Tốt nghiệp trường trung học cơ sở (Lớp 6-8)
- ☐ Một trường trung học phổ thông nào đó (Lớp 9-12)
- ☐ Tốt nghiệp trường trung học phổ thông (Lớp 9-12)
- ☐ GED/Chương trình tương đương trung học phổ thông
- ☐ Trường cao đẳng nào đó - không có bằng
- ☐ Bằng cao đẳng hoặc kỹ thuật (ví dụ: AA hoặc AS)
- ☐ Bằng đại học (ví dụ: BA, BS hoặc AB)
- ☐ Bằng cao học (ví dụ: Thạc sĩ hoặc Tiến sĩ)
- ☐ Không muốn trả lời

20. Hiện tại có bao nhiêu người sống trong hộ gia đình của quý vị? Tính cả bản thân quý vị, bất kỳ người lớn nào và bất kỳ trẻ em nào.

___ Người lớn (từ 18 tuổi trở lên) và ___ Thiếu niên (dưới 18 tuổi)

- ☐ Không muốn trả lời

21. Điều nào mô tả chính xác nhất tình trạng việc làm hiện tại của quý vị (chọn tất cả các mục phù hợp)?

- ☐ Đang làm việc bán thời gian (dưới 40 giờ một tuần)
- ☐ Đang làm việc toàn thời gian (40 giờ một tuần trở lên)
- ☐ Đang làm việc không lương (ví dụ: thực tập)
- ☐ Đang nghỉ làm (nghỉ thai sản)
- ☐ Không có việc làm và đang tìm việc
- ☐ Không có việc làm và KHÔNG đang tìm việc
- ☐ Đã nghỉ hưu
- ☐ Đang ở nhà, chăm sóc nhà cửa và người khác
- ☐ Không làm việc được vì bị tàn tật
- ☐ Đang đi học
- ☐ Khác ________________________________
- ☐ Không muốn trả lời

22. Quý vị có nói ngôn ngữ khác tiếng Anh tại nhà không?

- ☐ Có → **[NẾU CÓ]** Nếu có, ngôn ngữ nào?________________________
- ☐ Không
- ☐ Không muốn trả lời

23. Mức độ thường xuyên quý vị cần ai đó giúp đọc thông báo bằng văn bản từ bác sĩ hoặc hiệu thuốc là thế nào?

- ☐ Chưa bao giờ
- ☐ Hiếm khi
- ☐ Thỉnh thoảng
- ☐ Thường xuyên
- ☐ Luôn luôn
- ☐ Không muốn trả lời

Kết thúc khảo sát.

Nếu quý vị muốn được bốc thăm trúng thưởng một trong năm thẻ quà tặng Visa trị giá $50, vui lòng điền vào trang tiếp theo.

Q24: Nếu quý vị muốn được bốc thăm trúng thưởng một trong năm thẻ quà tặng Visa trị giá $50, vui lòng cung cấp tên, địa chỉ email hoặc số điện thoại của quý vị.

______________________________________ ______________________________________

Tên *Họ*

______________________________________ __ __ __- __ __ __- __ __ __ __

địa chỉ email *số điện thoại*
